# Decision criteria for the selection of analytical instruments used in clinical chemistry: IV External and internal evaluation of analytical instruments in clinical laboratory sciences

**DOI:** 10.1155/S1463924680000107

**Published:** 1980

**Authors:** M. Hjelm, T. D. Geary

**Affiliations:** 1 Department of Clinical Chemistry University Hospital Odense DK-5000 Denmark; 2 Division of Clinical Chemistry Institute of Medical and Veterinary Science Adelaide South Australia Australia


available, and many other factors. The list in Table 3 is by no means exhaustive and has been designed to facilitate the decision as to whether a given instrument is practicable under the conditions prevailing in the laboratory concerned.

These criteria cannot be assessed as objectively as those given previously.

### Conclusions

Consideration of the above criteria should make it possible to arrive at an objective decision at all times. However, it is essential that the following conditions are satisfied: (1) that suitable, quantifiable test parameters exist for these criteria; (2) that requirements are laid down for these test parameters, e.g. optimum values; and (3) that suitable data and information are available to cover the range of instruments from which selection is to be made. The conditions are far from satisfied and for this reason the IFCC Expert Panel on Instrumentation has a most important task – the evaluation and drafting of appropriate recommendations and standards.

REFERENCES

1. Commission on Automation, IUPAC Section of Clinical Chemistry. Characteristics and attributes of instruments intended for automated analysis in clinical chemistry. IUPAC Information Bulletin 1978, No. 3, 234-240.

2a. Büttner, J., Borth, R., Boutwell, J. H., and Broughton, P. M. G. (1978 b) Approved Recommendation on Quality Control in Clinical Chemistry. Part 1. General Principles and Terminology. Clin, Chim. Acta, to be published. Provisional Recommendation was published Clin. Chim. Acta **63**, F25 - F38 (1975).

b. Buttner, J., Borth, R., Boutwell, J. H., Broughton, P. M. G., and Bowyer, R. C. (1978 c). Approved Recommendation on Quality Control in Clinical Chemistry. Part 2. Assessment of Analytical Methods for Routine Use. Clin. Chim. Acta, to be published. Provisional Recommdendation was published Clin. Chim. Acta **69**, F1 - F7 (1976).

c. Buttner, J., Borth, R., Boutwell, J. H., Broughton, P. M. G., and Bowyer, R. C. (1977) Provisional Recommendation on Quality Control in Clinical Chemistry. Part 3. Calibration and Control Materials. Clin. Chim. Acta **75**, F11 - F20.

d. Buttner, J., Borth, R., Boutwell, J. H., Broughton, P. M. G., and Bowyer, R. C. (1978 a) Provisional Recommendation on Quality Control in Clinical Chemistry. Part 5. External Quality Control. Clin. Chim. Acta **83**, 191F -202F.

e. Buttner, J., Borth, R., Boutwell, J. H., Broughton, P. M. G., and Bowyer, R. C. (1977) Provisional Recommendation on Quality Control in Clinical Chemistry. Part 6. Quality Requirements from the Point of View of Health Care. Clin. Chim. Acta **74**, F1 - F9.

### Decision criteria for the selection of analytical instruments used in clinical chemistry

### IV External and internal evaluation of analytical instruments in clinical laboratory sciences

**M. Hjelm**

*Department of Clinical Chemistry, University Hospital, DK-5000 Odense, Denmark*

**and T.D.Geary**

*Division of Clinical Chemistry, Institute of Medical and Veterinary Science, Adelaide, South Australia*

THE performance and final choice of an analytical instrument are usually judged on two criteria, external and internal. The former are dependent on the experience of others, especially if the evaluation was carried out under conditions comparable to those in the purchaser's own laboratory. The latter relate to the purchaser's own assessment.

### The clinical requirements

There are, as yet, no "objective" rules for establishing clinical performance criteria for a particular biomedical analysis. Instead, it is frequently necessary to use allowable limits of error which are based upon present knowledge of the clinical requirements for systematic and random errors, while awaiting more accurate data arising from conferences. That held at Aspen in 1976 by the College of American Pathologists [1] forms a suitable guideline for these.

### External evaluations

These may take the form of published papers, reports given at regional, national and international meetings, or documents produced on behalf of or by various national, professional and governmental organisations. This information may be available from the manufacturer, but a list of evaluations has been published in the British Association of Clinical Biochemists' Newsletter, [2] and a revised list in a WHO Newsletter. It is the intention of the IFCC Expert Panel on Instrumentation to publish a revised list on a regular basis.

The Instrumentation Commission of the Clinical Biology Society of France produced a multi-centre evaluation protocol with which they assessed various enzyme rate analysers. This was based upon the National Comittee of Clinical Laboratory Standards Proposed Standard Evaluation Protocol - [1,3] and work by Broughton et al [4]. A working group of the German Society for Clinical Chemistry has taken this document and on behalf of the Expert Panel on Instrumentation of the IFCC, intend to prepare an evaluation procedure which should either be, or provide the basis for, an international standard. The final report prepared from data supplied by more than one laboratory will be less affected by the work patterns of each testing laboratory. It will therefore provide a strong basis for comparison.

**Multi-centre trial**

The proposed procedure will take the form of a multi-centre trial and include coverage of a range of parameters which are discussed in the following paragraphs.

*Technical evaluation*
This should take place before the analytical assessment and include electrical and mechanical safety, mechanical reliability of moving parts, physical characteristics, for example, of temperature control, stability against voltage variation and a review and audit of manufacturers claims concerning technical specifications.

*Analytical evaluation*
*Familiarisation protocol*
The design of this protocol will allow the laboratories concerned to become familiar with the operation of the instrument and to determine whether compatible data are being produced by all laboratories taking part in the trial.

*Full evaluation protocol*
This section of the analytical evaluation is commenced only if results from the technical and familiarisation evaluations are acceptable. It includes coverage of the following:

*Method dependent functions.* The performance of the instrument should be assessed by carrying out analytical procedures which challenge the various units, such as the pipetting mechanism, mixing device and spectrometer. The protocol includes provision for the testing of precision, accuracy, interferences, stability of reagents and drift, and provides sufficient data for the statistical analysis.

*Throughput.* An estimate of the time taken to analyse a sample and the maximum possible throughput per hour and day, taking into account standardisation and quality control procedures. Estimates would be made both when the instrument is fully operational and also when the instrument has to be started up.

*Precision.* A study of intra- and inter-batch replication of results should be made on samples with critical concentration(s) of analyte(s) and similar replicates from prepared coloured solutions. The design will provide information on the stability of reagents and drift.

*Accuracy.* The accuracy of results obtained should be compared against a reference or recommended method using materials with reference or consensus values. The effect of interferences on accuracy can be determined by testing related compounds and using haemolysed, icteric, or lipaemic sera.

*Criteria of acceptability.* The criteria of acceptability with regard to inaccuracy and imprecision should be agreed before any trial is undertaken.

*Method independent functions.* The protocol should be adapted to suit the system under assessment and may cover such functional modules as the signal measuring units, temperature control, volume dispensing devices and the effects of evaporation and contamination.

*Flexibility.* The flexibility of the system should be judged by considering such points as the possibility of using other chemical methods to measure the analytes, and other sources of reagents.

*Cost effectiveness (cost per test).* The cost of analysing 1, 20 or 50 samples, and of running the instrument at its maximum capacity should be determined.

*Operator and service manuals.* The quality of the instruction manual is a good guide to the quality of the instrument and to the service support available.

*Maintenance service.* A critical appraisal of the service which can be provided either by the manufacturer or the distributor is necessary.

*Review and audit of manufacturer's claims.* This should be undertaken using the guidelines such as those produced by the Expert Panel on Instrumentation [5,6] and by the American Association of Clinical Chemists for reagent kits [7].

**Internal evaluations**
When an assessment of the available instruments has been made by comparing the analytical and technical specifications with the results of any available external evaluations, an internal evaluation should be undertaken on the instrument of choice to determine whether it meets the laboratory's over-all requirements.

The scheme should include analysis of patients' sera with concentrations of the analytes covering the analytical range, together with a measurement of intra- and interbatch precision, and a review of the information provided by the manufacturer. If no report of a reliable external evaluation is available, as much as is practical of the previously suggested external evaluation procedure should be carried out.

### Summary
The schedules outlined above should provide the basis for the assessment of other instruments as well as chemical analysers. It is difficult to recommend specific procedural details in a testing approach which is intended for broad application, and when evaluating a specific instrument. The user will need to adapt the guidelines as necessary. The overall practicability of an instrument for any laboratory may be determined from the following: results of evaluations, required sample volume, technical time required, sample throughput, time for maintenance and cleaning procedures, the quality of the information provided in the service and operator manual, the after sales service backup, and cost effectiveness. It must be emphasized that if a laboratory's requirements are carefully defined beforehand, the final selection of the correct instrument will be greatly expedited and simplified.

REFERENCES

[1] *Analytical Goals*, College of American Pathologists, Aspen Conference 1976.
[2] *News Sheet* 174, Association of Clinical Biochemists, October 1977.
[3] National Committee for Clinical Laboratory Standards PSEP-1. *Protocol for Establishing Precision and Accuracy of Automated Analytic Systems..*
[4] Broughton, P. M. G., Gowenlock, A. H., McCormack, J. J. and Neill, D. W. *Ann. Clin.Biochem.*, (1974) **11**, 207.
[5] Okuda, K., Collombel, C., Geary, T. D., Haeckel, R., Mitchell, F. L. and Nadeau, R. *Clin.Chim.Acta.* (in press)
[6] Haeckel, R., Collombel.,C., Geary, T.D., Mitchell, F.L., Nadeau, R. and Okuda, K. *Clin.Chim.Acta.* (in press)
[7] American Association of Clinical Chemists. Policy regarding Reagent Sets and Kits. *Clin. Chem.*, (1966), **12**, 43

RECOMMENDED ADDITIONAL READING

National Committee for Clinical Laboratory Standards 771E. Lancaster Avenue, Villanova, PA 19085 USA
(1) Approved Standard ASI-I - Preparation of Manuals for Installation, Operation and Repair of Laboratory Instruments.
(2) Proposed Protocol PSEP-2 - Protocol for Establishing Performance Claims for Clinical Chemical Methods. Introduction and Performance Check Experiment.
(3) Tentative Standard TSC-5 - Methodological Principles for Establishing Principle Assigned Values to Calibrators.
(4) Tentative Standard TSI-3 - Standard for Determining Spectrophotometer Performance Criteria.
(5) Proposed Standard PSI-5 - Power Requirements for Clinical Laboratory Instruments and for Laboratory Power Lines.

